# Correlations of Geometry and Infill Degree of Extrusion Additively Manufactured 316L Stainless Steel Components

**DOI:** 10.3390/ma14185173

**Published:** 2021-09-09

**Authors:** Tobias Rosnitschek, Andressa Seefeldt, Bettina Alber-Laukant, Thomas Neumeyer, Volker Altstädt, Stephan Tremmel

**Affiliations:** 1 Engineering Design and CAD, University of Bayreuth, Universitaetsstr. 30, 95447 Bayreuth, Germany; bettina.alber@uni-bayreuth.de (B.A.-L.); stephan.tremmel@uni-bayreuth.de (S.T.); 2 Neue Materialien Bayreuth GmbH, Gottlieb-Keim-Str. 60, 95448 Bayreuth, Germany; andressa.seefeldt@nmbgmbh.de (A.S.); thomas.neumeyer@nmbgmbh.de (T.N.); volker.altstaedt@nmbgmbh.de (V.A.)

**Keywords:** material extrusion additive manufacturing, metal additive manufacturing, debinding and sintering, infill structure, effective porosity, product development

## Abstract

This study focuses on the effect of part geometry and infill degrees on effective mechanical properties of extrusion additively manufactured stainless steel 316L parts produced with BASF’s Ultrafuse 316LX filament. Knowledge about correlations between infill degrees, mechanical properties and dimensional deviations are essential to enhance the part performance and further establish efficient methods for the product development for lightweight metal engineering applications. To investigate the effective Young’s modulus, yield strength and bending stress, standard testing methods for tensile testing and bending testing were used. For evaluating the dimensional accuracy, the tensile and bending specimens were measured before and after sintering to analyze anisotropic shrinkage effects and dimensional deviations linked to the infill structure. The results showed that dimensions larger than 10 mm have minor geometrical deviations and that the effective Young’s modulus varied in the range of 176%. These findings provide a more profound understanding of the process and its capabilities and enhance the product development process for metal extrusion-based additive manufacturing.

## 1. Introduction

The social and environmental developments demand a more sustainable use of both available resources and products in their life cycle. These demands impose high challenges for the product development process. A strategy for more sustainable products is the exploitation of lightweight design potentials, which is, however, limited by conventional manufacturing technology [1]. Nature often uses various types of inner lattices to realize the potential of lightweight structures [2]. However, this approach can only be achieved by using additive manufacturing (AM). Due to its unique freedom of design and great material efficiency, AM can play a crucial role in future sustainable product development [1,3]. Since its beginning in the late 1980s, AM has widened its applicability from visual aids, design samples and technical prototypes to end-use parts. While looking explicitly at metal AM, the predominant processes for fabricating metal parts are powder bed fusion (PBF)-based, which has high investment costs for the machines and its peripherical equipment but also causes work safety issues due to the fine metal powder [4]. Thus, a promising alternative in some fields of application is the use of highly filled polymer filaments in extrusion-based additive manufacturing (EBAM), which are subsequently debinded and sintered, leading to nearly fully dense metal parts comparable to powder injection molded parts [5,6,7]. For this technique’s efficient and smart use, it is inevitable to use inner lattices to save material and fasten the fabrication process. Hence, most papers dealing with the material extrusion for metal parts are focused on material and process development [8,9,10,11,12,13,14]. It is necessary to investigate the impact of the infill degree on the mechanical properties and dimensional accuracy to enhance its capabilities as its product development process for further applications. Hence, it is the objective of this study to investigate the effective behavior and properties of metal EBAM parts for a given infill structure at various infill degrees to show the range of tunable effective part properties by applying different infill degrees.

### 1.1. Extrusion Based Additive Manufacturing of High-End Parts

A major disadvantage of EBAM is the relatively poor mechanical properties compared with conventional processes or PBF-based AM. Compared on an organizational level, EBAM machines are the most flexible and allow an exact prediction of the needed material since no reservoirs have to be filled. Additionally, EBAM processes are the most used AM processes ranging from hobby to industrial applications. Hence, it is favored to enhance the properties of EBAM parts to establish them in fields of application beyond prototyping. One strategy is to embed continuous fibers into the layer to create parts with much higher stiffness. The possibility to embed continuous fibers is thereby nearly exclusive for EBAM methods and is advantageous compared with other AM processes [15,16,17,18].

**Figure 1:**
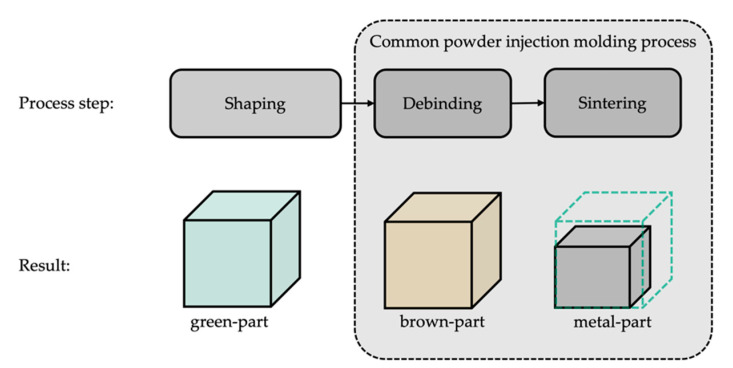
Schematic overview of the metEBAM process. The color scheme introduced for the parts will be used consistently in all figures below.

Another strategy is to produce metal or ceramic parts with highly filled polymers for subsequent debinding and sintering. This idea first appeared in the 1990s in the works [5,6,7] and was referred to as FDMet, an abbreviation for fused deposition modeling of metals, but further development was mainly hindered by blocking patents and difficulties in finding suitable binding systems [11]. The first commercially available filament for metal filament printing appeared in 2018 and was developed by BASF SE. Aside from BASF SE, Markforged Inc. and Desktop Metal Inc. have launched processes with EBAM-machines specifically for the fabrication of metal parts, naming their processes atomic diffusion additive manufacturing respectively bound metal deposition [19,20,21]. To avoid any conflicts with patented process names, the authors will refer to the technique as metal extrusion-based additive manufacturing (metEBAM). Nevertheless, the working principle of these processes, as illustrated in Figure 1, is more or less identical. In the first step, the green parts are shaped by an EBAM process. In the following, the debinding process removes the main component of the binding agent, leaving behind a porous brown part known from the field of powder injection molding (PIM), or more precisely for the particular case, metal injection molding (MIM) [22]. Subsequently, the parts are sintered in a furnace leading to a consolidation of the geometry, and a nearly fully dense metal part is obtained [11,12,23].

The process following the shaping step is analogous to the PIM route. Sintered metEBAM parts were studied for instance in [8,9,10,13,24,25,26]. For example, Damon et al. [8] reached a porosity of 0.5% and ultimate tensile strength of 500–520 MPa for 316L stainless steel while using completely filled specimens with rectilinear infill. Gong et al. [9] also conducted tensile testing of MetEBAM 316L and measured an ultimate tensile strength of 465 MPa and Young’s Modulus of 152 GPa at a porosity of 1.5%. Nevertheless, in [26] a significantly lower mean ultimate tensile strength of 311 MPa is reported. All of the previously mentioned studies conducted state that the non-uniform sinter shrinkage is one of the major concerns of the process that complicates the designing of metEBAM parts. Even though the general feasibility has been widely shown, for instance [13] already produced hollow profiles and a preliminary study of infill degrees has been made by [26], the systematic manufacturing of shelled parts with various infill degrees or the investigation of manufacturing complex parts have not been conducted yet to our knowledge.

### 1.2. Debinding and Sintering

Debinding and sintering are the two processes that transform the green-part into a metal-part and have accordingly high importance for the MetEBAM process. Residues of the binding agent have to be completely removed after debinding since carbon residues have a negative effect on the sintering behavior and the properties of the final metal part. Furthermore, a small backbone of the binding agent is required to ensure the part’s stability. Hence, the green parts are commonly debindered in two steps; in the first step, the main fraction of the binding agent is removed by catalytic or solvent debinding, and the residues are removed in the first stages of the sintering process by thermal debinding [11,12]. In general, catalytic debinding quickens the binder removal compared to solvent or thermal debinding and leads to higher brown-part strengths, which eases subsequent handling operations [12].

The subsequent sintering process involves several diffusion and rearrangement mechanisms, such as surface, lattice and grain-boundary diffusion, evaporation and condensation, and plastic and viscous flow [11]. For the sintering of 316L MIM parts, the results in [27] show that higher sintering temperatures lead to lower relative porosities and enhanced mechanical and corrosion resistance, accordingly. Further, the use of an Ar and H_2_ atmosphere showed low porosity and minimal fractions of O_2_ and N_2_ [27].

### 1.3. Structure-Property-Relations

The sintering process mainly affects the part’s microstructure. However, as part of the PIM process, the influence of sintering parameters on the microstructure is widely investigated in many industrial applications. As mentioned before, the axial shrinkage of the parts is deviant, while the total shrinkage correlates with the binding agent volume fraction [25]. In PIM, the exact sinter shrinkage is often analyzed experimentally [22]. Since PIM is a technology for very high volumes, this is not a good approach for the small batches in metEBAM. The researchers in [10] printed various features of interest, which could maintain their shape, showing that metEBAM parts can produce complex structures if the sintering shrinkage is compensated correctly. The work of [26] also analyzed the shrinkage behavior based on the part’s volume, height and surface area and noted a linear dependency between part dimension and shrinkage; for instance, flat printed tensile specimens were measured with a mean shrinkage of 16.57% in the *x-y* direction and 24.86% in the *z*-direction, although the coefficients of determination are relatively low. At the same time, the infill degree is reported to affect shrinkage behavior.

**Figure 2:**
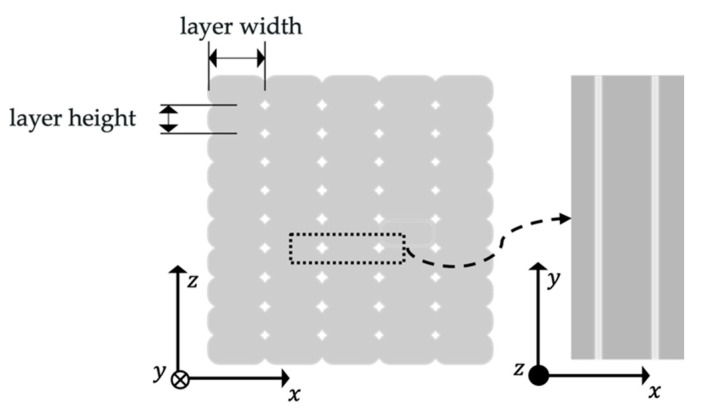
Schematical view of connected channel pores after sintering. The *z*-direction represents the stacking direction, while the *x-y* plane is the printing plane.

Nevertheless, the sintering shrinkage may become crucial during the design step to avoid internal stresses due to the large sinter-induced strains. The sintered microstructure, as shown for instance in [10], consists of small equiaxial grains with low porosity, indicating anisotropic behavior of the parts since the grains are not orientated as in the PBF processes. As shown in tensile tests [8,9,24,25], the specimens show anisotropic behavior. Following the work of Turner and Gold in [28], the metEBAM process coherently leads to voids between the single printing traces, causing a mesostructure of the green part dominated by the printing artifacts of the EBAM shaping. These voids cannot be closed during sintering, leading to connected pore-channels, as shown from CT-scans in [8], leading to a more or less periodically arranged network of porosity throughout the part, determining an orthotropic part behavior, Figure 2.

It is to conclude that both the part orientation and the filament deposition strategy are affecting its mechanical properties [29]. Nevertheless, the mechanical performance is comparable with PIM parts [8], which opens a potentially wide field of applications. Hence, it is inevitable to investigate the potential of the process to push its development further and start developing efficient methods for the related product development process. In previous work, the part and material properties have been investigated nearly exclusively for completely filled parts with rectilinear infill structures. Nevertheless, especially for lightweight engineering applications, it is of high interest to have insight into the effective properties for shelled parts with sparse infill structures, which have an intended effective porosity.

## 2. Materials and Methods

Within this article, we investigate the effective behavior of metEBAM austenitic stainless steel 316L components and focus on the part property correlations of geometry and infill degree in the meaning of mechanical behavior and dimensional deviations. We use a hexagonal honeycomb pattern as the infill structure and vary the infill degree levels to investigate the range of effective properties, which can be tuned by applying various infill degree levels.

### 2.1. Designing, Printing, Debinding and Sintering

**Figure 3:**
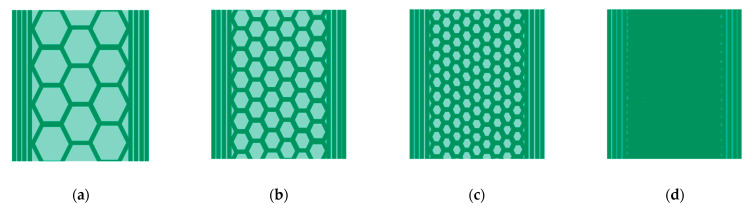
Sketch of the cross-sections of the used specimens. The outline of four layers remained constant while the size of the hexagonal infill structure was minified: (**a**) ID 25; (**b**) ID 50; (**c**) ID 75; (**d**) ID 100.

All parts in this study were printed with a shell of four outlines, four top and four solid bottom layers. The remaining volume was printed with a hexagonal honeycomb pattern as the infill structure at the infill degree levels of 25%, 50%, 75% and 100%, as illustrated in Figure 3. We will refer to these configurations later on as ID 25, ID 50, ID 75 and ID 100. To compensate sinter shrinkage, the dimensions in the printing plane are scaled by 1.2 and in the vertical direction by 1.26, as advised in the material data sheet [21].

#### 2.1.1. Material and Printing Parameters

The Ultrafuse 316L filament (BASF 3D Printing Solutions GmbH, Heidelberg, Germany) with an average diameter of 1.75 mm was used as received. The samples were manufactured using the GermanRepRap X500 Fused Filament Fabrication printer (innovatiQ GmbH + Co KG, Feldkirchen, Germany) with a copper nozzle having a diameter of 0.4 mm and the employed slicer was the commercially available Simplify 3D (Simplify3D, Blue Ash, Ohia, United States). The printing parameters were optimized in two sets of experiments while constantly maintaining the layer height of 0.2 mm, the printing speed of 25 mm/s and the nozzle temperature of 240 °C. The layer width was set to 0.5 mm for the practical experiments, as it was previously adjusted in the slicer to avoid voids.

The first set was performed to establish the optimal environment temperatures (printing bed and building chamber) to prevent warpage and ensure adhesion to the printing bed. The printing bed temperature was set to 90, 120 and 140 °C, while the tested building chamber temperatures were room temperature, 50 and 70 °C. The optimum layer width to guarantee the smallest distance between the layers was optimized in the slicer and varied until the result was qualitatively satisfactory. The extrusion coefficients tested were 1, 1.4, 1.6 and 2.

The technical datasheet specifies that a heated printing chamber is not required and that the temperature of the printing bed should be set between 90 and 120 °C [21]. However, severe warping was observed on the samples printed under these conditions, most probably due to the large surface of the specimens, as they have been printed in the *x-y* direction.

With printing bed temperatures of 90 and 120 °C, the specimens detached from the substrate and the printing process had to be stopped. Therefore, the optimum printing bed temperature, to ensure the proper adherence of the parts to the substrate during the entire printing process, was found to be 140 °C. In order to obtain warpage-free specimens, a print chamber temperature of 70 °C was also required. It is believed that temperatures higher than those indicated by the filament manufacturer were necessary due to the large surface area of the specimens in contact with the print bed since the tensile bars and bending specimens were printed in the *x-y* direction.

Different extrusion coefficients were tested in the second experiment set (1, 1.2. 1.6 and 2.0). The extrusion coefficient was increased because the material was visibly under-extruded with a typical coefficient of 1. Specimens printed with an extrusion coefficient equal to 2 showed deformations due to material excess. For this reason, only the specimens printed with extrusion coefficients from 1.2 and 1.6 were sent to the debinding and sintering steps. However, the parts produced with an extrusion coefficient of 1.2 collapsed during the sintering process, as there was not enough material to ensure the lateral overlapping of the layers. Therefore, the final testing specimens were printed using an extrusion coefficient of 1.6. Godec et al. have also observed that the quality of the printed parts using metal filed filament improves by increasing the flow rate [9]. This phenomenon could be associated with the high weight of the filament roll, making the material feeding more difficult. In addition, it could also be associated with the low viscosity and softness of the thermoplastic matrices used in metal-filled filaments, which also changes the filament feeding behavior. However, it is not possible to reach a definite conclusion with the data obtained so far; therefore, a more detailed investigation would be required.

The printing parameters used are listed in Table 1. Under these conditions, the printed parts were stable during further processing and did not present warping.

#### 2.1.2. Debinding and Sintering

After printing, the green parts were debindered and sintered at the facilities of BASF 3D Printing Solutions GmbH and Elnik System GmbH (Waldachtal, Germany). The Ultrafuse 316LX filament has a polyacetal binder system, and therefore, had to be processed using a catalytic debinding process, particularly BASF’s Catamold process [21]. In this particular case, the polyoxymethylene (POM) binding agent is catalytically dissolved at process temperatures in the range of 110–140 °C using HNO_3_ > 98.5%. The process temperature is significantly lower than the melting temperature of POM so that the polymer directly converses from solid to the gaseous state. The reaction interface between HNO_3_ and binder agent is moving inwards at approximately 2 mm/h; the resulting small formaldehyde gas molecules can escape the part through the porous outer zone without affecting its mechanical integrity [8]. After the main component of the binding system was removed, the obtained brown part consisted of approximately 2–3 wt.% of the residual binding agent [8]. This residual was needed so that the shape can be maintained. Then the first sinter-necks were formed during the primary sintering phase, the bounding of the metal particles was secured, and the residues of the binding system were removed thermally [12]. The sintering process was carried out in two steps in a hydrogen atmosphere. First, the parts were heated up at 5 K/min to 600 °C and held for 1 h to remove the binding agent residues. They were then heated up to 1380 °C and held for 3 h, where the actual sintering occurred, followed by a final cooling step [8].

### 2.2. Experimental Methods

#### 2.2.1. Dimensional Deviations

The dimensional deviations of the parts were measured manually for the bending and tensile specimens. The surface of the printed parts was not investigated further. The effective densities of the green and sintered parts were derived and compared with the filament and the bulk density of 316L, respectively, to determine the effective relative density of the parts, whereby we always referenced the density to the idealized solid geometry. The median and mean values with their standard deviations are presented in the following for all results.

#### 2.2.2. Tensile Testing

The tensile testing was performed according to DIN EN ISO 6892-1 procedure B [30]. Six specimens for each infill degree were tested on a universal testing machine Zwick 1485, with a loadcell of 250 kN (ZwickRoell GmbH & Co. KG, Ulm, Germany). The stress velocity in the elastic range was set to 10 MPas^−1^.

**Figure 4:**
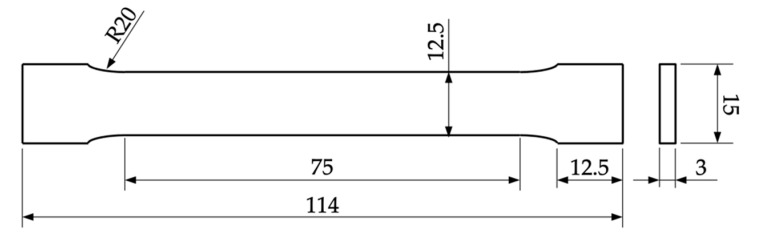
Tensile specimen with a total length of 114 mm and a test length of 75 mm. The specimens were printed horizontally so that the height correlated with the stacking direction.

After the yield stress was detected, the traverse speed was fixed at 0.008 s^−1^. The Young’s modulus was determined as the secant modulus in the range of 0.05% and 0.015% technical strain. The geometry of the tensile specimen is shown in Figure 4. As a consequence of the infill structure, as depicted in Figure 3, the specimens do not have a constant cross-section under loading. Since the objective of the experiment is the effective macroscopic part-behavior, we use the effective specimen cross-section width of 12.5 mm and height of 3.0 mm to calculate the stresses for all specimens.

#### 2.2.3. Bending Testing

**Figure 5:**
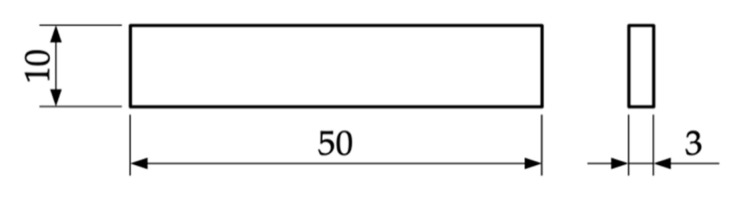
Bending specimen with length 50 mm, width 10 mm and height 3 mm. The specimens were printed horizontally so that the height correlated with the stacking direction.

The bending testing was also performed with six specimens for each infill degree on a universal testing machine Inspekt table blue (Hegewald & Peschke Meß- und Prüftechnik GmbH, Nossen, Germany). The procedure was according to EN ISO 3325 [31]. An outer fiber strain of 12.0% was chosen as the stopping criteria. The geometry of the bending specimen is shown in Figure 5. Since the specimens are loaded perpendicular to the infill structure, the loaded cross-section is constant.

## 3. Results and Discussion

### 3.1. Dimensional Deviations

**Figure 6:**
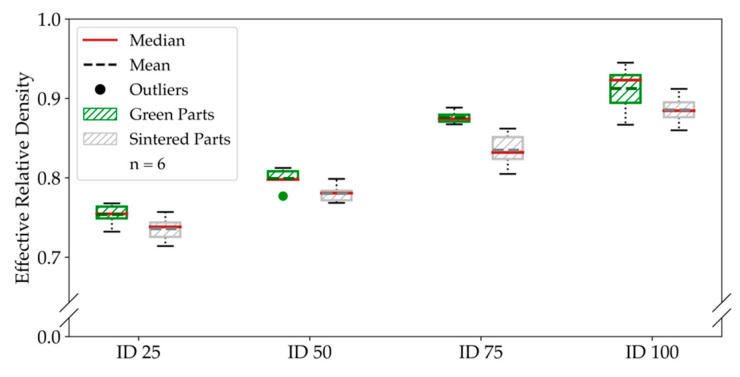
Effective relative density of the bending specimens. The comparison of the density before and after sintering shows a decrease in the relative density for the sintered parts.

The bending and tensile specimens were measured and weighed before and after sintering. Hence, we calculated the relative densities of the specimens and compared them before and after sintering. Figure 6 shows the comparison of green and sintered effective relative densities for the bending specimens.

**Figure 7:**
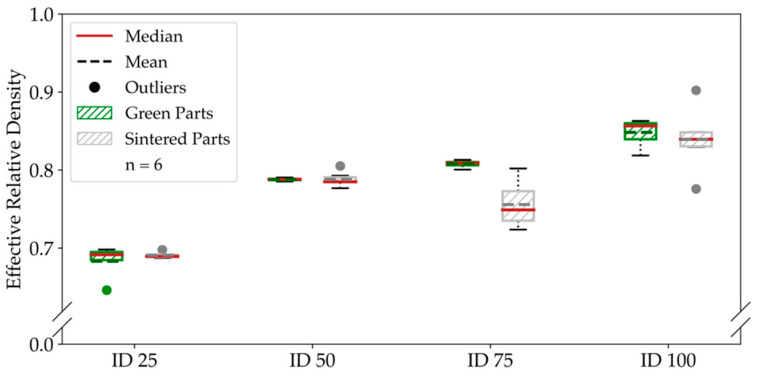
Effective relative density of the tensile specimens. The comparison of the tensile specimens’ density before and after sintering shows a constant relative density for ID 25 and ID 50 and a decrease for ID 75 and ID 100.

After sintering, we could save about 25% of weight by applying an infill degree of 25%, which corresponds to 75% of porosity in the bulk volume without considering the shell. The mean green-part effective relative density for completely filled bending specimens was 91.24%. For the sintered, completely filled ID 100 specimens, we could achieve a mean effective relative density of 88.55%. Consequently, the effective relative density decreased by around 3% during the sintering. For ID 25 and ID 50, the effective relative density decreased by approximately 1.8%. In total, we observed a mean decrease of effective relative density of 2.61% for the bending specimens. While the effective relative density decrease was very similar for all infill degrees of the bending specimens, the tensile specimens show much smaller changes for ID 25 and ID 50 than for ID 75 and ID 100, respectively, which we show in Figure 7. Furthermore, we reached a mean effective relative density for completely filled specimens of 84.82% before sintering.

In contrast to the bending specimens, the number of outliers after sintering increased for the tensile specimens. We reached a mean effective relative density for the completely filled ID 100 tensile specimens of 83.92%, a decrease of 5% compared to the bending specimens.

By setting the infill degree to 25%, we could reduce the mass by 31%. On the contrary, the density decrease during sintering is nearly 50% smaller for the tensile specimens, with a mean density decrease of 1.31%. In the case of the ID 25 tensile specimens, the effective relative density increased by 0.79% during sintering.

**Figure 8:**
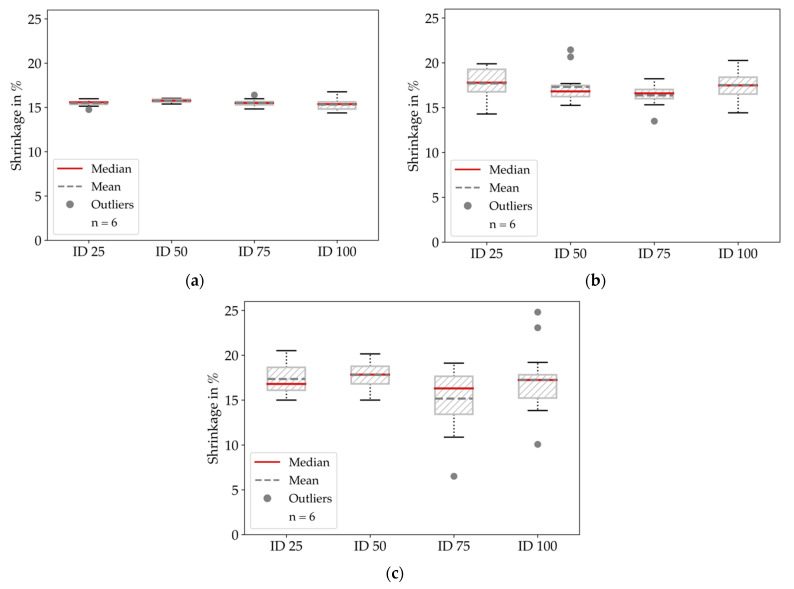
Global shrinkage behavior for all specimens and infill degree configurations: (**a**) Length (inplane, *x*-axis); (**b**) Width (inplane, *y*-axis); (**c**) Height (stacking direction, *z*-axis). The results show the slightest deviations for the length dimension.

Derived from the measurements before and after sintering, we summarized the global shrinkage behavior of all specimen configurations in Figure 8 and Table 2, where the length and width indicate the shrinkage in the printing plane and height indicates the shrinkage in the stacking direction.

The measured shrinkage for the height and width dimensions was about 2% greater than for the length dimension. Hence, the results indicate a lower shrinkage along larger dimensions. In contrast to the results in [8,10] or the datasheet information [21], we could not observe a significantly larger shrinkage in the stacking direction.

**Figure 9:**
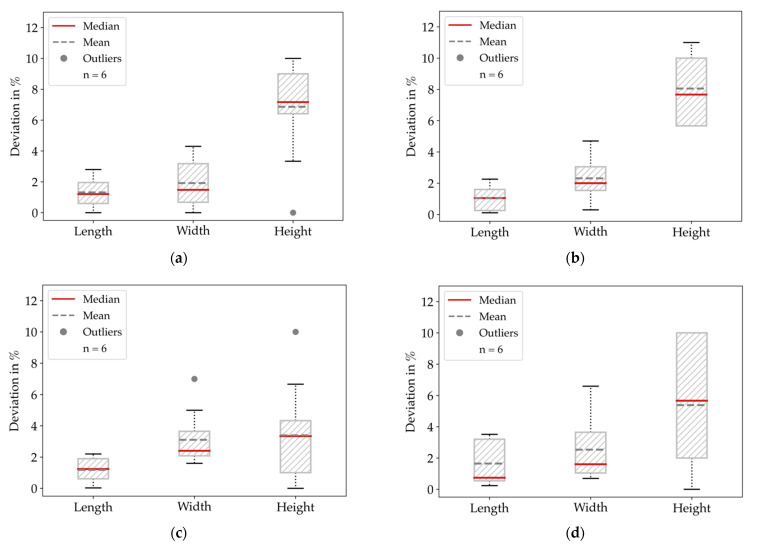
Global deviations between the CAD dimensions and the measured specimens: (**a**) ID 25; (**b**) ID 50; (**c**) ID 75; (**d**) ID 100. The smallest deviations are observed for ID 75.

Since we worked with the proposed scaling factors, which correspond to a shrinkage of 16% in the printing plane and 20% in the stacking direction, we got a bias in evaluating the deviations between the real parts and the dimensions of the CAD model. This bias for the shrinkage was below 2% in the printing plane and between 2.2% and 4.84% in the stacking direction. We show the global deviations between printed and sintered specimens and dimensions of the CAD model in Figure 9 and summarize the mean values in Table 3.

Considering the scaling factor bias, the deviations in the printing plane were within a range of 4.06%, whereas the height in the stacking direction deviated up to 6.86%. In summary, the accuracy was best for large dimensions and worse for smaller features.

### 3.2. Mechanical Testing

#### 3.2.1. Tensile Testing

**Figure 10:**
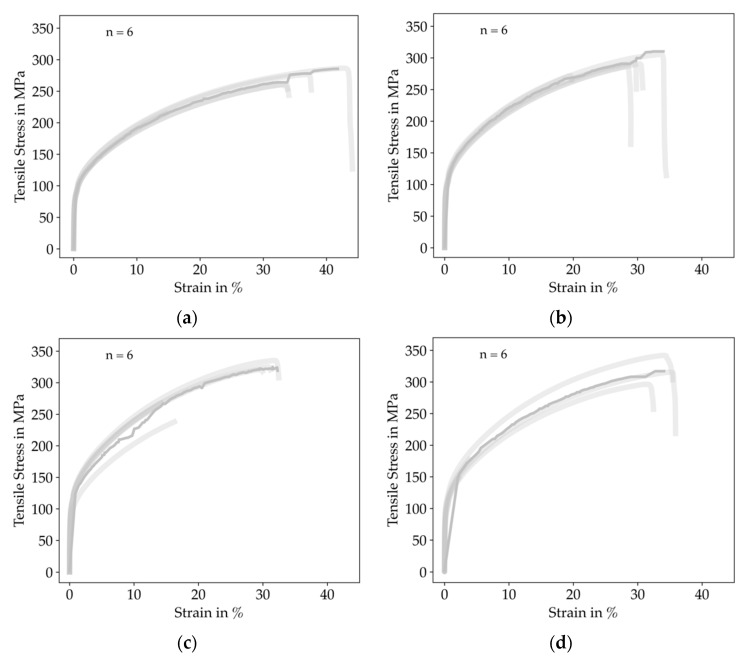
Resulting stress-strain curves of all tensile specimens clustered by infill degree. The solid line represents a mean-curve computed by rigid regression, the shaded lines represent the single measurements: (**a**) ID 25; (**b**) ID 50; (**c**) ID 75 and (**d**) ID 100. High deviations for the elongation at break were observed for all specimens.

The stress-strain curves from tensile testing for all specimens are plotted in Figure 10.

**Figure 11:**
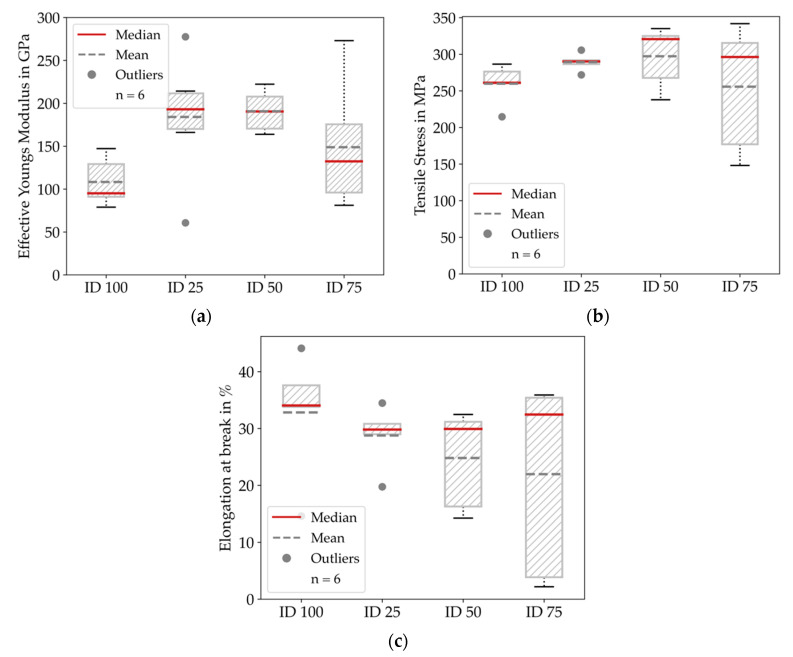
Results of the tensile testing experiments clustered by infill degree: (**a**) Effective Young’s modulus for all configurations; (**b**) Tensile stress for all configurations; (**c**) Elongation at the break for all configurations. The results show high deviations between the specimens, we could also tune the effective Young’s Modulus within the range of 108 to 191 GPa.

It is notable that the strain variance was significantly high for all configurations. We give a more detailed insight in Figure 11, where the effective Young’s Modulus, tensile stress and elongation at break for all configurations are summarized.

We only observed outliers for ID 25 and ID 50, which we attribute to the influence of the specimens’ non-constant cross-section. Regarding the effective Young’s modulus, ID 50 and ID 75 showed the highest values, and the mean for the lowest filled ID 25 and the completely filled ID 100 were significantly lower. Surprisingly, we also measured some specimens with superelevated effective Young’s modulus. We attribute these measurement errors to inaccuracies in the clamping of the specimens or the setting behavior of the crosshead measurement system.

Furthermore, the mean and median of the tensile stress were increasing with increasing infill degree up to ID 75 and then decreased for ID 100. Comparing the elongation at break, the mean and median were relatively constant for all configurations, with slightly higher values at ID 25 and ID 100. Nevertheless, the deviation between the maximum and the minimum value for ID 25 was 303% and 1645% for ID 100, respectively.

**Figure 12:**
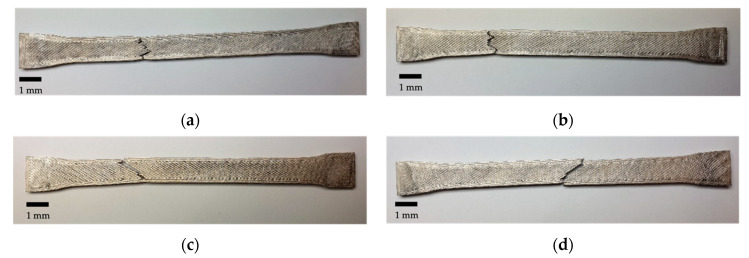
Fracture surfaces of the tensile specimens: (**a**) ID 25; (**b**) ID 50; (**c**) ID 75; (**d**) ID 100.

By evaluating the fracture surfaces presented in Figure 12, we observed that for ID 25 and ID 50, the specimens broke approximately in a straight line. Conversely, ID 75 and ID 100 broke at circa 45 degrees to the load direction, which indicates the highest normal stresses at 45 degrees and points to shear-stress failure. Additionally, the cracks follow the deposition path of the top- and bottom-layer, which could favor the dislocations. The infill patterns of ID 75 and ID 100 are small enough (Figure 3) to allow crack propagation at 45 degrees, whereas in ID 50 and ID 25, the crack propagation is deflected by the honeycomb pattern.

To set these results into a wider context, we show a comparison of ID 100 with other MetEBAM, PIM and selective laser melting (SLM) manufactured 316L stainless steel samples in Table 4.

This comparison shows that the elongation at break is comparable to other MetEBAM studies. The other effective properties of ID 100 are inferior compared with completely filled MetEBAM parts with rectilinear infill, MIM or SLM parts.

#### 3.2.2. Bending Testing

**Figure 13:**
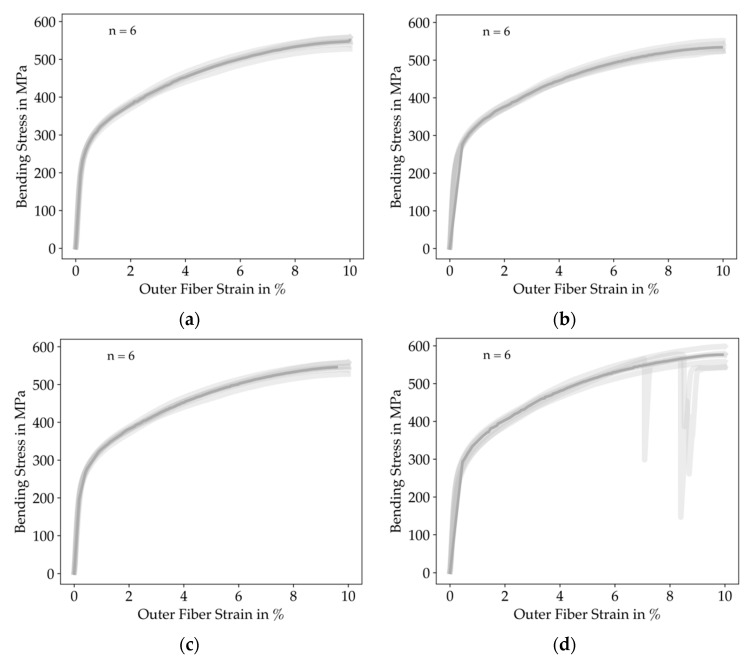
Resulting stress-strain curves of all tensile specimens clustered by infill degree. The solid line represents a mean-curve computed by rigid regression, the shaded lines represent the single measurements: (**a**) ID 25; (**b**) ID 50; (**c**) ID 75; (**d**) ID 100. The down-peaks in the ID 100 measurements were caused by the slipping of the specimens on the machine’s support. In general, the deviations between the various infill degrees are small.

We present the stress-strain curves of all tested bending specimens in Figure 13.

**Figure 14:**
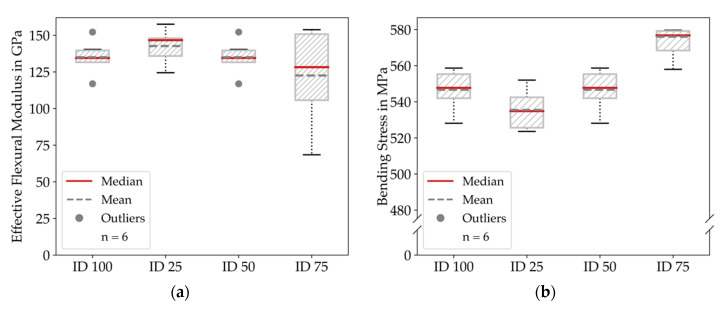
Results of the bending testing experiments clustered by infill degree: (**a**) Effective flexural modulus for all configurations; (**b**) Bending stress for all configurations. The flexural modulus is quasi constant for all infill degrees.

From the results, it is observable that the variance for each infill degree was smaller than for the tensile testing. The down-peaks in the ID 100 measurements occurred because the specimens slipped on the machine’s support. We give a greater insight into the results by comparing the flexural modulus and the bending stress for each infill degree in Figure 14.

Accordingly, to the median of the effective flexural modulus results, all configurations showed similar behavior with an effective flexural modulus ranging from 130 to 142 GPa; nevertheless, the variance was significantly larger for ID 100. Regarding the bending stress, the results were slightly lower for ID 50 and were slightly higher for ID 100 but also here, the deviations between the various infill degrees were below 10%.

## 4. Discussion

### 4.1. Dimensional Deviations

In terms of maximum effective relative density, we could achieve a maximum of 88.55% post-sintering, which is lower than, for instance, the results presented in [26] with 95.4% or [8] with 98.5%. We attribute this difference mainly to the hexagonal infill structure, which generally shows lower densities than the rectilinear infill structure used in [8,26]. In terms of effective properties, we can therefore conclude that the effective relative density for completely filled MetEBAM parts with hexagonal infill structure is significantly lower than for rectilinear filled. On the other hand, the model of the machine used, as well as the different printing profiles, may influence the effective relative density. Other machines might show a better accuracy in general that will lead to fewer process-induced pores.

By comparing the geometry of the specimens, we observed that by changing the geometry from bending to a tensile specimen, which is basically done by adding a section with another cross-section and applying a rounding feature to an initial rectangle, the effective relative density was reduced by 5%. This indicates that for more complex geometries, the maximum achievable density might not be comparable to the results for simple specimen geometries.

The shrinkage of the specimens did not show the assumed anisotropic behavior, which can be explained by the printing profile used, as the extrusion factor of 1.6 increases the amount of material extruded for one given layer. Nevertheless, this extrusion factor extrapolation was necessary for obtaining stable and high-quality specimens. To elaborate here, that for the given equipment, the extrusion factor equal to 1.6 assures high-quality samples while you are over-extruding your samples. According to this, our scaling factor for the green parts, which we chose beforehand, was not correct, resulting in a bias for calculating the geometrical deviations. Nevertheless, we observed that the geometrical deviations of the specimens were best for larger dimensions; in particular, for dimensions greater than 10 mm, we observed a deviation of below 2%. For geometrical features smaller than 5 mm, we observed deviations of about 4.5%. This can be explained by the fast acceleration required from the print head for smaller features, resulting in inaccuracy on the material deposition. Therefore, we recommend a feature size of about 10 mm for good results.

The effective relative porosity could be varied between 73.57% and 88.55% for bending specimens and between 69.02% and 83.92% for bending specimens, respectively. A clear link to the chosen infill degree is neither found for shrinkage nor for geometrical deviations; in general, we observed slightly better results for ID 50 and ID 75. Additionally, it is advisable to evaluate shrinkage specimens based on the used machine and printing profile to obtain a more precise scaling factor for the green parts and avoiding a scaling factor bias.

An interesting subject of future work will be the extent to which these results are transferable to more complex parts, for instance, topology optimized chassis diverters, as presented in [35]. Approaches for a physical simulation of the debinding and sintering process, which may eliminate the trial-and-error phase during part design, will play a key role for future applications of metEBAM. Such approaches are heavily needed since current approaches, for instance, as presented in [29], presume a pre-known shrinkage and simulate the sintering process on a phenomenological basis.

### 4.2. Mechanical Properties

Regarding the tensile testing results, we attribute the variance for ID 25 to ID 75 mainly to the influence of the infill structure on the loaded cross-section. Especially the deviations for the elongation at break can be seen as a consequence of the non-constant cross-section. One will assume that the effective Young’s modulus and tensile stress are increasing with an increasing infill degree; despite ID 50 and ID 75 showing quite identical behavior while ID 100 showed decreased values. We assume that the ID 100 specimens had a significantly higher amount of printing defects, as well as thermal tension accumulation due to the massive amount of material used, which impeded the homogenous cooling of the parts. This combination of effects affected the part’s mechanical properties. Therefore, the measured effective Young’s modulus describes the effective behavior of the shelled parts. Nevertheless, we could tune the effective Young’s modulus within the range between 108 and 191 GPa by using 25% to 75% infill degrees, respectively. This finding potentially allows the effective Young’s modulus to vary within a range of about 176% within one manufacturing step by applying various infill degree levels. Conversely, the mass of the parts could be reduced by over 20% without a significant loss of mechanical performance. Compared with more mature manufacturing technologies, the mechanical properties of metEBAM parts are still inferior, which can be linked mainly to process-induced pores, which lead to the low density of the filled specimens after sintering. With improved machine equipment, metEBAM is likely to be equivalent to MIM properties within the near future.

The bending testing results showed relatively minor deviations regarding the effective flexural modulus and bending stress. The tendency of the effective flexural modulus aligns with the effective Young’s modulus results with, in this particular case, slightly lower values for ID 25 and ID 100, and respectively higher moduli for ID 50 and ID 75. In general, these testing results can be attributed to the fact that most of the load is mainly absorbed by the shell layers, as it is known from sandwich structures.

Accordingly, the infill degree only has a minor influence on the part’s bending properties but plays a more significant role in tensile loading. The next step in this context will be to investigate the infill degree’s influence on the fatigue behavior of the parts.

Therefore, the effective Young’s modulus can be varied in a wide range without impacting the bending stiffness significantly. Furthermore, the mass can be reduced by over 20% without losing much of the mechanical performance, which is promising for use in lightweight engineering applications.

Interesting subjects for future work will be to investigate various wall and roof and floor layer combinations, as well as different infill structures. The characterization of effective properties of various shells, infill degrees and infill structures will be helpful for running parameter studies on effective part behavior to determine an optimal combination of shell, infill degree and infill structure for a given application.

## 5. Conclusions

This study aimed to investigate the part-property correlations of geometry and infill degree in the meaning of effective mechanical behavior and dimensional accuracy. The results show that it is possible to reduce the infill degree to a certain percentage (50% and 75%) without losing effective mechanical performance. On the contrary, they also indicate that completely filled specimens might show an increased tension accumulation and printing defects leading to inferior mechanical properties. It was also observed that dimensions larger than 10 mm showed about 50% minor geometrical deviations. Therefore, we suggest avoiding completely filled parts for hexagonal infill structures, as well as too small features. In terms of mechanical behavior, it is shown that the specimens behave similar to sandwich structures under bending loading and that the Young’s modulus can vary in a wide range. The latter leads to a wide range of applications in various engineering fields where the effective material behavior can be adjusted easily by using multiple infill degrees.

## Figures and Tables

**Table 1:** Final printing parameters.

Printing Parameter	Value
Nozzle temperature	240 °C
Chamber temperature	70 °C
Printing bed temperature	140 °C
Printing speed	25 mm/s
Layer height	0.2 mm
Layer width	0.5 mm
Extrusion factor	1.6

**Table 2:** Mean values of the sinter shrinkage for all configurations. *x*-axis and *y*-axis represent the printing plane, and *z*-axis represents the stacking direction.

	Length (*x*-Axis)	Width (*y*-Axis)	Height (*z*-Axis)
ID 25	15.48%	17.70%	17.35%
ID 50	15.75%	17.31%	17.80%
ID 75	15.52%	16.37%	15.16%
ID 100	15.31%	17.52%	17.26%

**Table 3:** Mean values for the deviations between the final part and CAD model. Length and width represent the printing plane and height of the stacking direction. The deviations adjusted for the scaling factor bias are given in brackets.

	Length Deviation	Width Deviation	Height Deviation
ID 25	1.31% (0.79%)	1.92% (2.62%)	6.86% (4.21%)
ID 50	1.04% (0.79%)	2.31% (3.62%)	8.06% (6.86%)
ID 75	1.18% (0.70%)	3.31% (3.67%)	3.39% (−1.45%)
ID 100	1.65% (0.96%)	2.54% (4.06%)	5.39% (2.65%)

**Table 4:** Comparison of metEBAM produced 316L stainless steel with PIM and SLM material properties. It is to note that the median values of ID 100 were taken from the tensile specimens, additionally, the standard deviation is given in brackets.

Mechanical Property	ID 100	PIM [32]	SLM [33]	AISI 316L [34]	MetEBAM [8]	MetEBAM [10]
Tensile strength in MPa	296 (78)	557	644	515	500–520	465
Elongation at break in %	32 (16)	30	51	60	32–37	31
(effective)Young’s modulus in GPa	132 (65)	no data	165	193	185	152
(effective sintered) relative density in %	83.9 (3.7)	92.1	99.9	100	99.5	98.5

## Data Availability

The data presented in this study are available on reasonable request from the corresponding author.

## References

[1] Tofail S.A.M., Koumoulos E.P., Bandyopadhyay A., Bose S., O’Donoghue L., Charitidis C. (2018). Additive Manufacturing: Scientific and Technological Challenges, Market Uptake and Opportunities. Mater. Today.

[2] Clausen A., Andreassen E., Sigmund O. (2017). Topology Optimization of 3D Shell Structures with Porous Infill. Acta Mech. Sin..

[3] Bourell D.L. (2016). Perspectives on Additive Manufacturing. Annu. Rev. Mater. Res..

[4] Scime L., Wolf S.D., Beuth J., Mrdjenovich S., Kelley M. (2018). Safety and Workflow Considerations for Modern Metal Additive Manufacturing Facilities. JOM.

[5] Agarwala M.K., Jamalabad V.R., Langrana N.A., Safari A., Whalen P.J., Danforth S.C. (1996). Structural Quality of Parts Processed by Fused Deposition. Rapid Prototyp. J..

[6] Wu G., Langrana N.A., Rangarajan S., McCuiston R., Sadanji R., Danforth S.C., Safari A.A. Fabrication of Metal Components Using FDMet: Fused Deposition of Metals. Proceedings of the Solid Freeform Fabrication Symposium.

[7] Wu G., Langrana N.A., Sadanji R., Danforth S.C. (2002). Solid Freeform Fabrication of Metal Components Using Fused Deposition of Metals. Mater. Des..

[8] Damon J., Dietrich S., Gorantla S., Popp U., Okolo B., Schulze V. (2019). Process Porosity and Mechanical Performance of Fused Filament Fabricated 316L Stainless Steel. Rapid Prototyp. J..

[9] Godec D., Cano S., Holzer C., Gonzalez-Gutierrez J. (2020). Optimization of the 3D Printing Parameters for Tensile Properties of Specimens Produced by Fused Filament Fabrication of 17-4PH Stainless Steel. Materials.

[10] Gong H., Snelling D., Kardel K., Carrano A. (2019). Comparison of Stainless Steel 316L Parts Made by FDM-and SLM-Based Additive Manufacturing Processes. JOM.

[11] Gonzalez-Gutierrez J., Cano S., Schuschnigg S., Kukla C., Sapkota J., Holzer C. (2018). Additive Manufacturing of Metallic and Ceramic Components by the Material Extrusion of Highly-Filled Polymers: A Review and Future Perspectives. Materials.

[12] Rane K., Strano M. (2019). A Comprehensive Review of Extrusion-Based Additive Manufacturing Processes for Rapid Production of Metallic and Ceramic Parts. Adv. Manuf..

[13] Rane K., Cataldo S., Parenti P., Sbaglia L., Mussi V., Annoni M., Giberti H., Strano M., Fratini L., di Lorenzo R., Buffa G., Ingarao G. (2018). Rapid Production of Hollow SS316 Profiles by Extrusion Based Additive Manufacturing. Proceedings of the 21st International ESAFORM Conference On Material Forming: ESAFORM, Palermo, Italy, 23–25 April 2018.

[14] Thompson Y., Gonzalez-Gutierrez J., Kukla C., Felfer P. (2019). Fused Filament Fabrication, Debinding and Sintering as a Low Cost Additive Manufacturing Method of 316L Stainless Steel. Addit. Manuf..

[15] Chacón J.M., Caminero M.A., Núñez P.J., García-Plaza E., García-Moreno I., Reverte J.M. (2019). Additive Manufacturing of Continuous Fibre Reinforced Thermoplastic Composites Using Fused Deposition Modelling: Effect of Process Parameters on Mechanical Properties. Compos. Sci. Technol..

[16] Czasny M., Goerke O., Kaba O., Koerber S., Schmidt F., Gurlo A. (2019). Influence of Composition on Mechanical Properties of Additively Manufactured Composites Reinforced with Endless Carbon Fibers. KEM.

[17] van de Werken N., Tekinalp H., Khanbolouki P., Ozcan S., Williams A., Tehrani M. (2020). Additively Manufactured Carbon Fiber-Reinforced Composites: State of the Art and Perspective. Addit. Manuf..

[18] Pezold D., Rosnitschek T., Kleuderlein A., Döpper F., Alber-Laukant B., Dröder K., Vietor T. (2021). Evaluation of Technologies for the Fabrication of Continuous Fiber Reinforced Thermoplastic Parts by Fused Layer Modeling. Technologies for Economic and Functional Lightweight Design.

[19] Desktop Metal Inc Desktop Metal Metal Filaments. https://www.desktopmetal.com/materials.

[20] Markforged Inc Markforged Metal Filaments. https://markforged.com/materials/.

[21] BASF 3D Printing Solutions GmbH BASF Metal Filaments. https://forward-am.com/material-portfolio/ultrafuse-filaments-for-fused-filaments-fabrication-fff/metal-filaments/.

[22] German R.M. (2012). Metal powder injection molding (MIM): Key trends and markets. Handbook of Metal Injection Molding.

[23] Rosnitschek T., Glamsch J., Lange C., Alber-Laukant B., Rieg F. (2021). An Automated Open-Source Approach for Debinding Simulation in Metal Extrusion Additive Manufacturing. Designs.

[24] Gonzalez-Gutierrez J., Arbeiter F., Schlauf T., Kukla C., Holzer C. (2019). Tensile Properties of Sintered 17-4PH Stainless Steel Fabricated by Material Extrusion Additive Manufacturing. Mater. Lett..

[25] Jimbo K., Tateno T. (2019). Shape Contraction in Sintering of 3D Objects Fabricated via Metal Material Extrusion in Additive Manufacturing. Int. J. Autom. Technol..

[26] Ait-Mansour I. (2020). Design-Dependent Shrinkage Compensation Modeling and Mechanical Property Targeting of Metal FFF. Prog. Addit. Manuf..

[27] He H., Li Y.M., Li D.P. (2011). Effect of Sintering Temperature and Atmosphere on Corrosion Behavior of MIM 316L Stainless Steel. AMR.

[28] Turner B.N., Gold S.A. (2015). A Review of Melt Extrusion Additive Manufacturing Processes: II. Materials, Dimensional Accuracy, and Surface Roughness. Rapid Prototyp. J..

[29] Rosnitschek T., Hueter F., Alber-Laukant B. (2020). FEM-Based Modelling of Elastic Properties and Anisotropic Sinter Shrinkage of Metal EAM. Int. J. Simul. Model..

[30] DIN EN ISO 6892-1:2019 (2019). Metallic Materials—Tensile Testing—Part 1: Method of Test at Room Temperature.

[31] DIN EN ISO 3325:1999 + A1:2002 (2002). Sintered Metal Materials, Excluding Hardmetals—Determination of Transverse Rupture Strength.

[32] Hausnerova B., Novak M. (2020). Environmentally Efficient 316L Stainless Steel Feedstocks for Powder Injection Molding. Polymers.

[33] Röttger A., Boes J., Theisen W., Thiele M., Esen C., Edelmann A., Hellmann R. (2020). Microstructure and Mechanical Properties of 316L Austenitic Stainless Steel Processed by Different SLM Devices. Int. J. Adv. Manuf. Technol..

[34] AISI Type 316L Stainless Steel Material Data. http://asm.matweb.com/search/SpecificMaterial.asp?bassnum=mq316q.

[35] Rosnitschek T., Hentschel R., Siegel T., Kleinschrodt C., Zimmermann M., Alber-Laukant B., Rieg F. (2021). Optimized One-Click Development for Topology-Optimized Structures. Appl. Sci..

